# *‘Mostly women's issues’* – Gender differences in community responses to a large-scale NGO programme to prevent violence against women in urban India

**DOI:** 10.1016/j.wsif.2024.102997

**Published:** 2024

**Authors:** Sukanya Paradkar, Chatush Singh, Anand Suryavanshi, Apurva Tiwari, Beniamino Cislaghi, Nayreen Daruwalla, David Osrin, Lu Gram

**Affiliations:** a Institute for Global Health, University College London, London, United Kingdom; b Society for Nutrition, Education and Health Action, Mumbai, India; c London School of Hygiene and Tropical Medicine, London, United Kingdom

**Keywords:** Community participation, Violence against women, Bystander action, Male engagement, Community health, South Asia, India, Informal settlements

## Abstract

Despite striking gender differences in men's and women's engagement in past prevention programmes to stop violence against women (VAW), few empirical studies have determined why such gender differences arise. We did a grounded theory study of a large-scale NGO programme in informal settlements in Mumbai, India, aiming to analyse how gender affects participation in community action to address VAW. We did 27 focus group discussions and 31 semi-structured interviews with 77 women and 36 men, as well as with 9 NGO staff. We supplemented qualitative data with quantitative monitoring data on referrals to NGO counselling centres. We found that male participants in the NGO programme not only reported violence to the NGO at lower rates but took less intensive action to support survivors. When they did engage, they more often defended perpetrators or asked survivors to accommodate them than female participants. These differences could be explained by a greater (1) affective response to VAW (2) perceived stake in addressing VAW (3) sense of empowerment from taking action, and (4) perceived NGO support for self among women compared to men. Differences were evident even between male and female participants who had taken part in NGO activities for years. We theorise that these differences ultimately stem from men and women's structurally different position in a gender unequal society. Our findings identify key motivational barriers to address to improve the effectiveness of programmes to prevent VAW. Given the greater barriers to men's community action compared to women's community action, it may be useful to recognize the value of prevention programmes with differing levels of male engagement, including women-only programmes and programmes in which male community participants play a secondary role and are not expected to be as active as female participants.

## Highlights

• We studied community responses to a program to prevent violence against women in India.
• We found marked gender differences in program participants' willingness to act.
• Men were much less willing to take action to stop violence than women.
• Gender differences were evident even among long-term participants.
• Men and women's structurally different position in society likely accounts for these differences.

## 1 Introduction

Violence against women (VAW) is a global public health crisis that violates human rights and carries severe human, emotional, and economic costs (Garcia-Moreno & Watts, 2011). VAW is widespread, with 27 % of women globally having experienced physical or sexual intimate partner violence in their lifetime (Sardinha et al., 2022). Policymakers, practitioners, and scholars increasingly recognize full community participation as necessary to ending VAW (Jewkes et al., 2021; USAID, 2020) and some of the most effective known examples of interventions to prevent VAW mobilise communities to address its social and structural drivers (Bourey et al., 2015; Keith et al., 2023). Interventions in the past have, for example, helped community members to raise awareness of the problem, intervene in cases of violence, and refer survivors to community leaders (Lowe et al., 2022).

Globally, non-government organisations (NGOs) are important in addressing gender inequality, promoting women's empowerment, and preventing VAW in many contexts (Bernal & Grewal, 2014). In India, NGOs started occupying a niche in the national social welfare infrastructure in the post-liberalization era of the 1990s, when Nehruvian centralised socialist planning gave way to a development agenda shaped by global neoliberal ideas and policies (Gupta & Sharma, 2006). For example, NGOs in India play key roles in mobilising feminist activism, engaging communities in preventing VAW, and providing services to survivors of violence (Roychowdhury, 2016). At the time, NGOs were critiqued for “NGOizing” feminist praxis by professionalising and deradicalizing feminist social movements, thereby limiting their potential for transformative political change (Roy, 2015).

Engaging men and boys is argued globally to be a vital component of NGO and community efforts to end VAW (Jewkes et al., 2015). Whilst men are the majority perpetrators of VAW, many do not use violence and can be instrumental in persuading other men to change their behaviour (Tolman et al., 2019). However, men's engagement in the anti-violence movement has long been fraught with controversy, as it requires ‘mobilising a socially privileged group to work toward dismantling a problem largely perpetuated from within its own ranks’ (Casey et al., 2013) (p. 229). Concerns have been raised around male allies supplanting women's voices or leadership, men being cast as ‘saviours’ or ‘protectors’ of women, or men co-opting resources meant for female activists or survivors (Glick & Fiske, 2001; Jewkes et al., 2015).

Women's participation in NGO and community action to address VAW, by contrast, has often been seen as a means of female empowerment (Sanyal, 2009; Sweetman, 2013; Zulver, 2022). Solidarity with other women is argued to provide alternative social networks, freeing women from dependency on family and marital relations whilst enabling collective action to address gender injustice (Sweetman, 2013). For example, financial self-help groups for women in India and Bangladesh have enabled rural married women normally restricted by gendered mobility norms to break through social isolation, gain self-confidence, and acquire a degree of economic independence (Kabeer, 2011; Kumar et al., 2021; Sanyal, 2009).

Despite the potential to contribute empirically to the debate on men's role to prevent VAW, studies of NGO and community programmes have rarely analysed gender differences in action to address VAW. Those who have done this have found uneven levels of participation. Process evaluations of self-help group interventions in India reported that male engagement had ‘essentially failed’ (Jejeebhoy et al., 2017), as men lacked time or interest in violence prevention efforts (Holden et al., 2016; Jejeebhoy & Santhya, 2018). By contrast, an NGO-led community-based intervention in Uganda was found to nearly double rates of bystander action to address VAW among both men and women, with men in fact reporting higher levels of bystander action than women (Abramsky et al., 2018). These varied results demonstrate a need to explain and understand gender differences in action to address VAW as part of efforts to improve programmes and policies to prevent VAW.

We conducted a grounded theory study of community action to address VAW in a large-scale community programme in informal settlements in Mumbai, India, implemented by an NGO named SNEHA (Society for Nutrition, Education and Health Action). We conceptualise action to address VAW as a form of collective action (Lowe et al., 2022) which includes both primary prevention activities (such as awareness-raising campaigns) and secondary prevention activities (such as bystander action in response to witnessing violence). Our main research question was: how does gender shape community action to address VAW?

### 1.1 Prior studies of action to address VAW

Drivers of action to address VAW have been reported at multiple ecological levels. At the individual level, political psychologists have emphasised the degree of which action is seen as a legitimate response to the problem, an effective solution, and a reflection of social identity (Radke et al., 2016). At the community level, community psychologists have explored constructs such as critical consciousness, visibility of VAW as a public issue, capacity for informal social control, and community trust (Menon & Allen, 2020). Studies have also found material incentives for programme participation (or lack thereof) and institutional support to shape action (Gibbs et al., 2014; Hatcher et al., 2020; Stern et al., 2021). Social norms framing domestic violence as a ‘private issue’, risk of backlash, and presence of public crime have all been found to act as barriers (Gram et al., 2023). Increasingly, emotion is being recognized as an important driver of action as well (Gram et al., 2023). However, none of these studies have examined why or how gender differences arise.

Clues to gendered differences can be found by comparing studies that have analysed either men or women's participation in action to address VAW. For example, an analysis of 29 interviews with organisations engaging men in violence prevention across the world found male perceptions of anti-violence as a ‘woman's issue’ or inherently ‘anti-male’ to be common barriers (Casey et al., 2013). Studies of women's participation have reported critical consciousness, collective empowerment and charismatic leadership as enablers, and risk of backlash as a barrier (Hatcher et al., 2011; Sanyal, 2009; Zulver, 2022). These findings suggest that the same factors may motivate men and women to act, yet in different ways. For example, a systematic review found that women's and men's bystander action to address sexual assault in the United States were motivated by similar psychological influences, but compared to men, women had lower levels of inhibiting influences such as belief in rape myths or lack of self-efficacy (Labhardt et al., 2017).

### 1.2 Violence against women in Mumbai, India

India has ratified the Convention on the Elimination of All Forms of Discrimination Against Women (UN, 2008) and the Indian Penal Code and the Protection of Women from Domestic Violence Act of 2005 contain provisions addressing VAW. Nonetheless, the most recent estimates from informal settlements in Mumbai find that 13 % of women have experienced physical violence, 4 % sexual violence and 19 % emotional violence in the past year (Daruwalla et al., 2020). Women are assigned gender roles as primary caregivers for children and domestic violence is often justified as a punishment for transgressing gender norms (Visaria, 2000). Social norms curtail women's mobility and social interaction outside the home (Cislaghi et al., 2020) and street sexual harassment is common (Subbaraman et al., 2014; Zietz & Das, 2018).

### 1.3 SNEHA's violence prevention programme

SNEHA's violence prevention programme covers over 50,000 households and provides services to over 2000 survivors of violence annually in informal settlements in Mumbai, India. The NGO has a pro-feminist ideology in which women are seen as active agents in transforming their own and fellow community members' lives and men are potential feminist allies capable of questioning their own privilege and challenging gender injustice (Casey et al., 2018). Professional counsellors support survivors through counselling, referral to mental health professionals, and collaboration with medical, legal, shelter, and police services (Daruwalla et al., 2019). Such support takes a feminist, intersectional, and rights-based approach that respects survivors' agency in deciding on a course of action for themselves (Daruwalla et al., 2024).

Community engagement teams facilitate group meetings and community campaigns with women, men, and adolescents, which aim to raise critical consciousness in participants by encouraging reflection and critique of gender roles, gender inequality, and VAW. Group members are encouraged and supported by NGO staff to take collective action in their community to address both violence and non-violence related issues—such as provision of electricity, water, or sanitation services. In group meetings, NGO staff also provide participants with information on health, government entitlements, and legal rights. Group meetings with men particularly emphasise critical examination of gender roles and privileges through group discussion and debate, role play in which men are invited to put themselves in women's shoes and even wear women's clothes, and encouragement to take up domestic work responsibilities. Group meetings also focus on how the privileges held by men can be used positively within relationships instead of using subtle power and control.

Community identification and referral for survivors play a central role in SNEHA's theory of change (Daruwalla et al., 2019), which posits that secondary prevention—visibly responding to the needs of survivors—can act as a mechanism for primary prevention—preventing violent behaviour from arising in the first place. Male and female attendees at group meetings are encouraged to report cases of violence to SNEHA. The organisation selects particularly keen group members to become volunteers who are given extra training to identify, support, and refer survivors to NGO and government services. There are no explicit criteria for how group members are chosen to become volunteers. Staff make a subjective judgment based on a given group member's degree of initiative and willingness to lead, confidence in communication, social commitment, and interest in tackling VAW and participating in SNEHA activities.

Training sessions for volunteers aim to develop their understanding of VAW and develop their capabilities for action. Training sessions for male and female volunteers overlap in content, but differ in emphasis. Both male and female volunteers receive training on identifying and supporting survivors of violence and engaging with police, courts, and hospitals. However, female volunteers receive training on ‘barefoot counselling’ involving personally going and mediating in couple disputes and advising neighbours facing violence. Training for male volunteers places greater emphasis on questioning male privilege, gender socialisation, patriarchy, gender inequality, toxic masculinity, consequences of violence on interpersonal and family relationships and understanding allyship. Male volunteers receive training on allyship and are asked to organise the annual White Ribbon campaign, a male allyship campaign, in their neighbourhood. During this campaign, they serve as exemplars of positive deviance through actions such as combing their wife's hair or painting her nails on a stage in public.

Many group members, volunteers, and community members living in SNEHA's programme areas have been observed to be highly engaged in addressing VAW, often initiating or leading individual or collective action on their own in response to incidents of violence (Gram et al., 2024). Such responses can range from emergency intervention, such as physically interrupting ongoing violence, to emotional, social, and financial support for survivors, to verbal sanctions or threats against perpetrators, to referral to police and NGO services (Gram et al., 2024). Community members rely on their own creativity to devise strategies to respond to VAW, which may be inspired by content discussed in SNEHA group meetings, but also largely draw on own notions of appropriate responses to VAW (Gram et al., 2024). Whilst SNEHA staff sometimes play a role in guiding community members in action, the majority of actions taken by community members are initiated and led by themselves.

## 2 Methods

We conducted a grounded theory study (Corbin & Strauss, 2014) of gender differences in responses to VAW. Consistent with theoretical sampling, we alternated between data collection and data analysis. We continuously modified topic guides and sampling frames to explore new concepts identified during analysis. We supplemented qualitative data with quantitative data from SNEHA's monitoring database on referrals to its counselling centres, which enabled quantitative comparison of activity levels between men and women.

### 2.1 Qualitative data collection

#### 2.1.1 Sampling and recruitment

In line with theoretical sampling (Corbin & Strauss, 2014), we sampled respondents based on factors expected to influence their ability and motivation to address VAW to explore enablers and barriers to action. For this, we first stratified by individual exposure to the NGO programme, categorising respondents as either (1) a current member of an NGO group or (2) an NGO volunteer. Second, we identified neighbourhoods with NGO staff which had been particularly active or inactive in tackling VAW and sampled programme participants from them for focus group discussions (FGDs). Third, we sampled individual FGD participants for follow-up semi-structured interviews (SSIs). We sampled both male and female respondents to see if any findings relevant to action to address VAW would emerge from their comparison. Finally, to triangulate findings, we sampled NGO staff (counsellors, community organisers, programme officers, and a programme coordinator), residents who had ceased participating in NGO activities, and community members who had never participated in NGO activities. NGO staff recruited participants in person or over the phone. NGO staff carrying out recruitment were distinct from NGO staff who acted as respondents themselves.

We conducted 6 FGDs with 31 men, 16 FGDs with 75 women, 8 SSIs with men, and 19 SSIs with women (Table 1, Table 2). We conducted follow-up FGDs and SSIs with 3 groups of women and 3 women interviewees to elaborate on key points from earlier discussions. We also conducted five FGDs and four SSIs with nine NGO staff members. NGO staff members faced a degree of difficulty in gaining access to male community members. Male respondents generally preferred online to face-to-face interviews and needed multiple calls and reminders to be available for interview. However, apart from two male group members who declined to participate in SSIs due to lack of time, no other respondents refused.

**Table 1:** List of focus group discussions with community members.

ID	Sex	Type	Expected activity level	Number of participants	NGO exposure
FG1	Female	General	N/A**	5	None
FG2	Female	General	N/A**	7	None
FG3	Female	Group members	Active	6	4 years
FG4	Female	Group members	Inactive	6	3–5 years
FG5	Female	Group members	Active	5	3 years
FG6	Female	Group members	Inactive	5	3 years
FG7	Female	Group members	Inactive	6	2–4 years
FG8	Female*	Group members	Active	8	3 years
FG9	Female	Volunteers	Active	7	1–15 years
FG10	Female	Volunteers	Active	5	8–14 years
FG11	Female	Volunteers	Inactive	4	3–7 years
FG12	Female*	Volunteers	Inactive	5	3 years
FG13	Female*	Volunteers	Active	6	1–6 years
MG14	Male	General	N/A**	5	None
MG15	Male	General	N/A**	5	None
MG16	Male	Group members	Active	4	4 years
MG17	Male	Group members	Inactive	7	1.5 years
MG18	Male	Volunteers	Active	5	2–13 years
MG19	Male	Volunteers	Inactive	5	1.5–4 years

*Note. ‘*Volunteers’ are NGO-trained community volunteers. ‘Group members’ participate in NGO-run group meetings in the community. ‘General’ refers to general community members who are neither volunteers nor group members. ‘Expected activity level’ refers to the degree to which NGO staff judged the group to be actively engaged in tackling VAW—rather than passive—at the stage of sampling. * Received a follow-up focus group discussion. ** NGO staff cannot judge activity levels of community members who do not join NGO events.

**Table 2:** List of semi-structured interviews with community members.

ID	Sex	Type	Age	Marital status	Religion	Education	Occupation	Interview mode	Years of NGO engagement
FI1	Female	General	26–35 years	Married	Muslim	5th grade	Homemaker	Face to face	NA
FI2	Female	General	56–65 years	Widowed	Muslim	12th grade	Homemaker	Face to face	NA
FI3	Female	General	56–65 years	Married	Muslim	<5th grade	Stitching work	Face to face	NA
FI4	Female	General	26–35 years	Married	Hindu	4th grade	Homemaker	Face to face	NA
FI5	Female	Group member	36–45 years	Married	Muslim	None	Homemaker	Face to face	4 years
FI6	Female	Group member	36–45 years	Married	Hindu	8th grade	Electrical parts	Face to face	5 years
FI7	Female	Group member	36–45 years	Married	Hindu	10th grade	Homemaker	Face to face	3 years
FI8	Female	Group member	36–45 years	Married	Muslim	<5th grade	Homemaker	Face to face	3 years
FI9	Female	Group member	36–45 years	Married	Hindu	12th grade	Teacher	Face to face	4 years
FI10*	Female	Group member	36–45 years	Married	Muslim	<5th grade	Homemaker	Face to face	3 years
FI11	Female	Volunteer	36–45 years	Married	Muslim	8th grade	Homemaker	Face to face	15 years
FI12	Female	Volunteer	36–45 years	Married	Hindu	10th grade	Beauty parlour	Face to face	3 years
FI13	Female	Volunteer	36–45 years	Married	Muslim	8th grade	Homemaker	Face to face	2 years
FI14*	Female	Volunteer	46–55 years	Married	Hindu	7th grade	Shop owner	Online	6 years
FI15	Female	Volunteer	26–35 years	Married	Muslim	5th grade	Stitching work	Online	3 years
FI16	Female	Volunteer	36–45 years	Married	Muslim	7th grade	Homemaker	Face to face	7 years
FI17*	Female	Volunteer	36–45 years	Married	Hindu	8th grade	Stitching work	Face to face	15 years
FI18	Female	Volunteer	36–45 years	Married	Muslim	<5th grade	Homemaker	Face to face	4–5 years
FI19	Female	Ex-volunteer	20–25 years	Married	Muslim	9th grade	Homemaker	Face to face	1.5 years
MI20	Male	General	20–25 years	Unmarried	Hindu	12th grade	Sales	Online	NA
MI21	Male	General	36–45 years	Married	Muslim	5th grade	Welding	Online	NA
MI22	Male	Group member	20–25 years	Unmarried	Hindu	Bachelor's	Banking	Online	4 years
MI23	Male	Group member	26–35 years	Married	Muslim	8th grade	Transport	Online	3 years
MI24	Male	Volunteer	46–55 years	Married	Hindu	10th grade	Driver	Face to face	13 years
MI25	Male	Volunteer	20–25 years	Unmarried	Buddhist	Bachelor's	Municipal worker	Face to face	10 years
MI26	Male	Volunteer	26–35 years	Unmarried	Hindu	Bachelor's	Unemployed	Online	3 years
MI27	Male	Ex-volunteer	56–65 years	Married	Buddhist	9th grade	Garments	Online	15–16 years

*Note. ‘*Volunteers’ are NGO-trained community volunteers. ‘Group members’ participate in NGO-run group meetings in the community. ‘General’ refers to general community members who are neither volunteers nor group members. * Received a follow-up interview.

#### 2.1.2 Procedure and materials

Data collection took place from 2021 to 2022 across two large informal settlements in Mumbai. Author SP conducted all the FGDs and SSIs in Hindi or Marathi. Topic guides for community members broadly focused on perceived enablers and barriers to action to address VAW. We elicited respondent attitudes to VAW, asked about specific incidents of violence and their responses, and explored the perceived roles of public institutions in tackling violence. Topic guides for NGO staff covered overall NGO strategy to prevent VAW, the perceived role of community members in this strategy, and NGO relations with communities.

FGDs and SSIs were conducted either online over Google Meet (meet.google.com), over the phone, or face-to-face depending on the preferences of participants and lasted 30 to 90 min. All FGDs and SSIs were audio-recorded. Two translators transcribed 10 % of recordings into Hindi or Marathi before translating them into English. SP directly translated the remaining recordings into English. Author LG listened to recordings and read through transcripts in Hindi and English to check translation quality. SP and LG held debriefing sessions after each FGD and SSI to discuss new concepts and plan further data collection. SP and LG iteratively adjusted topic guides based on ongoing data analysis.

### 2.2 Quantitative data collection

We extracted quantitative data from SNEHA's monitoring database on referrals to counselling centres situated in the two large informal settlements from which we sampled our qualitative respondents. Counsellors registered new clients into an online database hosted by CommCare (www.dimagi.com/commcare) using a tablet-based interface. Upon registration, counsellors indicated whether the client was referred by a female community volunteer, a male community volunteer, a female member of a SNEHA group, a male group member, or someone else (hospital staff, SNEHA community staff, family member, etc.). Counsellors could also select ‘self-referral’ if clients had come on their own initiative.

We computed an indicator of male and female programme participants' activity levels by calculating the average number of referrals made by male group members, female group members, male volunteers, and female volunteers. Though community action to address VAW takes other forms besides reporting cases (Gram et al., 2024), we chose referrals as an marker of participant activity levels due to the central role played by identification, referral, and support for survivors in the organisation's theory of change (Daruwalla et al., 2019). We calculated numbers from 2018 through to 2022. We disaggregated by financial year, as SNEHA records numbers of group members and volunteers by financial year.

### 2.3 Data analysis

SP and LG coded transcripts of FGDs and SSIs. We used the software tool MAXQDA 2020. We memoed transcripts using analytical tools from grounded theory, followed by open, axial and selective coding (Corbin & Strauss, 2014). We developed separate coding trees for men's and women's responses to incidents of VAW and enablers and barriers to action to address VAW. We constantly compared coding trees in terms of similarities and differences between men and women. SP and LG discussed interpretations and findings with each other, co-authors, and NGO staff almost daily to ensure analytic rigour, stimulate reflective analysis, and reduce cultural bias. SP also called past interviewees on the phone to clarify statements. We did a descriptive quantitative analysis by comparing referral-to-programme participant ratios by gender and type of participant (group member or volunteer). We held a meeting with senior and junior NGO staff to discuss findings and receive feedback on data analysis.

### 2.4 Researcher positionality

Researchers' social position, experiences, and beliefs can critically influence research processes and findings (Berger, 2015). As such, we provide a brief statement of positionality. Three authors (including first author SP) identify as female, four (including last author LG) as male. Four (including SP) are employees of SNEHA based in Mumbai, whilst three (including LG) are academics based in the United Kingdom. Being a local Indian qualitative researcher and PhD student in political science, SP had a somewhat privileged socioeconomic background compared to local respondents. However, social distance between SP and respondents was muted by her youth given powerful hierarchies of age in communities. LG had a higher degree of privilege, being male and based in a high-income country. LG sought to counteract this by drawing on his extensive experience living and working in South Asia, analogies to own lived experiences of racism, and epistemic humility in dialogue with local partners. The study itself directly addressed a key desire by SNEHA to better understand the mechanisms of its violence prevention programme.

### 2.5 Ethics

Ethical approval for the study was provided by Partners for Urban Knowledge, Action, and Research (PUKAR) in India (30 June 2021), and the University College London Research Ethics Committee (16603/001, 20 June 2021). We followed World Health Organisation guidelines for research on domestic violence against women (WHO, 2001). Respondents were given participant information sheets and asked for consent. For face-to-face interviews, we obtained signed consent, or thumb prints when respondents could not read or write. For online interviews, we took verbal consent. Explicit text in information leaflets and consent forms stating that community members' decision to participate did not in any way affect their access to SNEHA's services for VAW was read aloud and explained to all participants. Participants were required to confirm on the consent form that they understood this point before agreeing to participate. A protocol was followed for action in cases of disclosure of abuse or signs of distress from participants, including referral to services for survivors of violence.

## 3 Results

### 3.1 Gender differences in action to address VAW

FGDs and SSIs with male and female respondents indicated qualitative differences in action to address VAW. Male and female participants had both supported survivors. However, men tended to prefer indirect forms of action which minimised their own involvement, such as calling the police anonymously or knocking on doors and leaving to interrupt couples fighting. Several men even cast standing around and watching during a fight as a major intervention: “*If one person just stands there, then 50% of the issue gets sorted out*” [MI18]. Although both men and women expressed a need to investigate ‘who was right’ in disputes before intervening, men frequently framed this in a defensive way, stressing the possibility of men's innocence: “*I understood that it's futile to make a decision based on hearing only one side. Both sides should be heard … It is not true that men are always wrong, never women.*” [MI23]. When male participants had involved themselves more closely, this had resulted in problematic actions. One volunteer had denied allegations from his friend's wife of domestic abuse, claiming that she attempted suicide by drinking poison simply to frame his friend [MG19]. Another had suggested to a distraught neighbour whose husband was wasting money on alcohol and bargirls to get him drunk and dance for him like a bargirl to entice him to drop money in her lap [MG18]. This prompted an NGO staff member who worked with men in the local community, including the participant in question, to interrupt him and, surprisingly, admonish him. This did not seem to make a difference, as the participant maintained his view for the rest of the FGD, but we also explained to the staff member not to repeat this behaviour in the future.

Female participants were willing to entangle themselves in fights between couples and did not report actions that supported or accommodated perpetrators. Participants had offered to mediate in disputes, escorted survivors to the police to demand action, and even confronted perpetrators and threatened them with social or legal repercussions. “*I tried to reason with him. He said, ‘Ok, I won't do it [beat his wife] again.’ I said, ‘If I see you doing something like again, there won't be anyone worse than me. You don't know what I can do!*’” [FI8]. They had supported survivors in myriad ways, such as stopping suicide attempts, bringing survivors home to their parents, taking them to hospital, or offering financial help or shelter, and had had detailed and intense discussions with survivors about their experiences of violence.

Quantitative data broadly supported qualitative findings that female participants were more engaged than male participants (Table 3). 52 % of referrals to SNEHA's counselling services were due to female programme participants, whilst 2 % were due to male programme participants. The number of survivors referred to SNEHA per female volunteer ranged from 0.80 to 0.91 in 2019–2022. The number of referrals per male volunteer ranged from 0.13 to 0.66 in the same period. Comparing group members, female group members referred 0.16–0.38 survivors per group member in 2019–2022. Male group members almost never referred cases to SNEHA, having referred only 0.02–0.05 survivors per group member in the same period.

**Table 3:** Cases of violence against women referred to SNEHA's counselling services.

	2019–20	2020–21	2021–22
Number of participants			
Female volunteers	390	398	333
Male volunteers	40	50	40
Female group members	1610	1735	1475
Male group members	185	160	143

Number of referrals			
Self-referrals	113	103	79
Female volunteers	312	345	303
Male volunteers	5	33	20
Female group members	435	667	232
Male group members	7	8	3
SNEHA staff	255	173	109
SNEHA hotline	2	380	110
Other institutions (police, hospitals, NGOs)	147	126	69
Other	134	215	59

Referral-to-participant ratio			
Referrals per female volunteer	0.80	0.87	0.91
Referrals per male volunteer	0.13	0.66	0.50
Referrals per female group member	0.27	0.38	0.16
Referrals per male group member	0.04	0.05	0.02

Note. Years provided are financial years (e.g. 2019–20 runs from 1st April 2019 until 31st of March 2020), in accordance with SNEHA's accounting system for programme participants.

### 3.2 Gender differences in enablers and barriers to addressing VAW

Respondent accounts highlighted several reasons for gender differences in levels of activity. We have thematically organised these below.

#### 3.2.1 Affective response to VAW

Female respondents frequently expressed strong affect in discussions on VAW. Some described past incidents of VAW that they had witnessed, which had left them so upset, sad, or anxious that they were compelled to act. For example, some female volunteers described ‘feeling like crying’ or welling up, listening to survivors: “*She was so upset, even my eyes welled up looking at her! How she was bearing all this, suffering so much!*” [FG9]. Others were clearly frustrated and outraged on behalf of survivors. Women's descriptions of abusers contained a condemnatory tone that was almost entirely absent in interviews with men. Perpetrators were described as ‘evil’, ‘characterless’, ‘bastards’, ‘demons’, ‘devils’, or ‘rapists.’ For example, a female group member described some perpetrators as incorrigibly evil: “*Those filled with shamelessness and have all kinds of filth inside them, they will keep bringing forth filth. Whatever happens, even if they are beaten because of their behaviour, even if they are beaten to death, they will remain lost to humanity.*” [FG3].

Male participants—even those who had taken part in NGO activities for up to thirteen years—tended to engage with the issue of VAW at a depersonalised level, framing the need to tackle it as a corollary of general moral imperatives to respect the rights of other people. The affective content of most their accounts was more muted and involved descriptors of violence such as ‘uncomfortable’, ‘weird’, and ‘troubling’, rather than words describing anger or upset. For example, a volunteer said that it was in his nature to do ‘*social work*’, like having an ‘*itch*’ that needed scratching [MI24]. In contrast to female respondents who almost always brought up own family issues, male respondents almost never discussed gender roles, power dynamics, or violence in their own family. Instead, they often diverted discussions onto other topics, such as local investment schemes, or compared action to address VAW to general social obligations:

*We volunteers don't just stand and watch [violent incidents], but we ask, “What's the matter?” We try to work things out. For example, during the rains, there was a problem with an uncovered manhole and potholes had also formed. No-one was doing anything. I complained to the municipal office! … My neighbourhood, my city, and also my country is important to me.*

[FGD with male volunteers, MG18]

#### 3.2.2 Perceived stake in addressing VAW

Female respondents broadly believed that tackling VAW advanced their interests and improved their circumstances. In cases of sexual harassment, child molestation, or murder, the offender constituted a clear risk to respondents themselves. In cases of domestic violence, many saw their own experiences reflected in those of survivors. Some were themselves survivors or had witnessed abuse in childhood. Where they had formerly felt powerless to tackle violence in their own lives, they now felt vicariously empowered by supporting others. Others were frustrated with women's status in society and saw prevention efforts as contributing to broader shifts in power between men and women:

*There are men who think that they should try to keep a woman down as much as they can. They think that women don't have the right to speak, to raise their voice … [But] a woman, if needed, can also become a Goddess of Destruction [literally ‘a Kali or a Durga’].*

[FGD with female volunteers, FG10]

Male respondents for the most part did not see efforts to address VAW as something of direct benefit to themselves. Prevention activities were done ‘for women's sake’ [MI23] and VAW was mostly a ‘women's issue’*.* Frustrated with being asked to reflect on VAW, a male community member even exclaimed, “*See, this issue is about women! And about this, only those men who live here with their family can tell you about it. We don't know much about this.*” [MG15]. Even after probing by the interviewer, the other FGD participants remained silent, perhaps indicating tacit agreement. In another FGD, male participants went as far as to claim that men were blamed unfairly for violence caused ‘primarily’ by women, “*All these incidents [of VAW] that are happening, 100% of the time, it is caused by women … Women are the ones who weaken each other, and the man becomes the bad guy in all this.”* [MG18]. Some men were also uncomfortable with the need to challenge their powers and privileges as men. A male volunteer noted that participants had once objected to role playing as women in a five-day workshop on gender roles. When asked the next day if they would listen to a female head of household, they said, “*This doesn't exist anywhere! … We don't give women that kind of status!”* [MI25].

#### 3.2.3 Sense of empowerment

Women often said that they felt more knowledgeable, confident, and independent from participating in efforts to address VAW. Many felt they had gained courage from leaving the confines of their home, participating in public NGO activities, and experiencing solidarity with other women. Many described learning to ‘stand on their own two feet’, ‘rise to the occasion’, or draw on their inner strength to tackle problems. Some had even surprised their family members as they started standing up for themselves, answering back, and arguing their own case. For example, a volunteer noted, “*They [family members] say, ‘Look! She's become smart! Earlier, when she was gullible, it was better. Now, that she's meeting people, with SNEHA coming and giving her new information, she's smarter.’*” [FI17]. Some female volunteers had become informal community leaders who were routinely approached by neighbours to resolve family disputes. One volunteer exclaimed, “*In the beginning, nobody responded to us, nobody knew about us, nobody would even speak to us … Now, the entire area is ours!*” [FI17].

Male respondents—for the most part—did not report a sense of empowerment from efforts to address VAW. Men's accounts tended to display a tone of sombreness and dutifulness, having learned the changes required in their own and other men's behaviour to bring about a better society, including becoming more respectful and less judgmental of women. For example, a volunteer even said, “*Earlier I used to think that my problems were a lot, but now my issues are nothing and there are bigger problems in society*” [MG19]. Strong taboos on social contact between men and women also created ubiquitous fears that men who intervened in incidents of VAW would be accused of inappropriate relations with survivors. For example, a man had wanted to confront a stranger who was sexually harassing a woman on his street, but his wife warned him against intervening in case neighbours mistook him for being the harasser instead [Interview with NGO programme coordinator]. An NGO staff member received complaints from the community early in their career for simply approaching local women to speak to them about SNEHA's work on domestic violence [Interview with NGO staff]. Men ended up asking female volunteers to accompany them to acquire legitimacy when speaking to survivors.

#### 3.2.4 Perceived NGO support

Female respondents ascribed their sense of empowerment in large part to support from SNEHA. By throwing its organisational weight behind poor women and educating women in responding to VAW, SNEHA inspired and galvanized women. As an external organisation employing educated staff versed in the law, SNEHA commanded a status that ordinary community members did not have. For example, a community member said that perpetrators would never listen to neighbours, but “*no matter how far a person has fallen, he'll still listen to [SNEHA], because they are external.*” [FI3]. Residents name-dropped SNEHA as a partner organisation to push for action by police or hospital staff and lobbied SNEHA for physical ID cards to prove their association to a public body. Female participants also felt that successful resolution of cases of violence caused neighbours to take notice: “*People who joined SNEHA initially … did not know what SNEHA was. Gradually, over the last three years, their lives changed, at home and in public. The abuse, they faced stopped. Then people understood that SNEHA is something.*” [FI7].

Male participants had mixed views. All welcomed SNEHA's presence and expressed gratitude for education and support they had received. However, male respondents, female respondents, and NGO staff all raised concerns about perceptions of SNEHA as ‘biased’ in favour of women. One male volunteer said, “*We've been taught that SNEHA is only for women and children. Men have no role here. They have no authority here … That is what we've been taught for the 12 years that I've been here.*” [MG18]. These perceptions were difficult to challenge, as they drew on powerful community narratives that public institutions provided special treatment to women. For example, a male community member interpreted the fact that a local NGO (different from SNEHA) facilitated police action in domestic violence cases as ‘injustice’ and ‘giving all the power to women’, because the man was being questioned for his actions despite fulfilling traditional gender role obligations as provider for the family [MG14]. This caused misunderstandings, as respondents believed that SNEHA only listened to women's side of disputes, did not hold women accountable, or provided more support to female than male volunteers. Some even complained that SNEHA provided female, but not male volunteers with ID cards, whereas in fact SNEHA did not issue ID cards to any volunteers [MG18].

## 4 Discussion

We analysed impacts of gender on participation in community action to address VAW in an NGO programme in Mumbai, India. Male programme participants reported cases of VAW to SNEHA at considerably lower rates than female programme participants, and action taken by male participants was often less intensive than action by female participants. Male participants also took problematic forms of action We found four major reasons for these differences, stemming from differences in men's and women's: (1) affective response to VAW, (2) perceived stake in addressing VAW, (3) sense of empowerment from taking action to address VAW, and (4) perceived NGO support. Surprisingly, these differences were evident even among male and female participants who had engaged in NGO activities for years. Our findings suggest that addressing these important factors—including the role of emotion (León & Montenegro, 1998; Gram et al., 2023)—may improve the effectiveness of community programmes and renew questions on the role of men in VAW prevention, given the greater barriers to men's community action in our context.

We broadly theorise that NGO efforts to raise consciousness in a context of wider structural gender inequality produce discrepancies in men and women's willingness to take action to address VAW. At root, gender inequality shapes social structures and social norms in our context in well-documented ways: women face risks of gender-based domestic and public violence (Visaria, 2000; Zietz & Das, 2018), restrictive gender norms limit women's mobility and ability to socially interact with non-kin men (Cislaghi et al., 2020; Gram et al., 2023), and men are generally privileged in private and public institutions (Ahmed-Ghosh, 2004; Phadke et al., 2011). At the same time, past evidence indicates that SNEHA's efforts at raising consciousness activities do make many programme participants and their neighbourhoods more aware of women's rights and more committed to stopping VAW (Gram et al., 2024). We hypothesise that the interaction between SNEHA's efforts to raise consciousness and the social and cultural context produce differential effects on male and female programme participants that overall favour women's rather than men's community action to address VAW.

Our theory explains how the comparatively greater barriers to men's participation in action to address VAW may stem from structural differences in men and women's social position. Flood (2015) notes that men stand to lose very real patriarchal privileges from ending VAW, even if progress towards gender equality is not “a zero-sum game in which men will lose and women will gain” (p. 167). As a sign of the sense of threat this engenders, in India as elsewhere in the world, men's rights organisations have emerged as part of a wider patriarchal backlash against women's anti-violence advocacy (Lodhia, 2014). Conversely, women's lived experience of low status in public and private life with attendant risks of violence (Visaria, 2000; Zietz & Das, 2018) clearly facilitates an emotional connection to the issue. In prior research, we have noted how men face barriers to identifying and empathising with women's struggles due to their peer networks being dominated by other men (Gram et al., 2023). All of this makes it more difficult to motivate men than women to see themselves as having a stake in stopping VAW or to elicit affective responses among men compared to women.

Social norms constraining women's public presence are increasingly in flux (Daruwalla et al., 2017), which created opportunities for SNEHA to break with norms and engender a sense of empowerment through activities such as women's group meetings and leadership training for female volunteers. Taboos on social contact between the sexes—which rendered men vulnerable to accusations of improper conduct when intervening in cases of violence—were conversely entrenched. Empowering men was challenging, because they are already privileged in certain respects: they do not face social norms limiting their mobility outside the home and often have more opportunities than women for status advancement and material reward through participation in private and public bodies such as political parties. For example, a household survey found that men in our context were 1.4 times more likely than women to participate in community organisations, and more than twice as likely to engage with political, caste, or religious issues, if they did so (Gram, Chakraborty, et al., 2021a).

Contesting perceptions that SNEHA was ‘biased’ in favour of women was also difficult, as they were likely not simply borne out of misperceptions of SNEHA's mandate, but also awareness of the material fact that the majority of NGO services—whether maternal and child health education, female livelihoods training, or counselling for survivors of violence—did respond to desires and needs more often expressed by local women than men. The fact that this was interpreted as indicating ‘bias’ in favour of women in a way reflected male privilege, as there were plenty of institutions (police, political parties, religious associations, etc.) in which men had a leading role and it was precisely this patriarchal default—such as police and justice systems turning away domestic violence survivors to ‘protect the family’ (Sukhtankar et al., 2022)—which necessitated a response to women's needs in the first place. However, it also meant that SNEHA could not simply correct misperceptions by implementing extra services for men to ‘balance out’ its portfolio, as this might deepen gender inequalities, whilst superficially appearing to promote equality (MacKinnon, 2011).

We caution that our findings should not be interpreted to mean that all community women became empowered through engagement with SNEHA or that no men were interested in VAW prevention. We had male respondents who were keen on VAW prevention and female respondents who did not engage at all. Gender roles and norms are contingent and shifting (Daruwalla et al., 2017) and direct comparison between men and women runs the risk of essentialising a fixed gender binary. Men and women are not homogeneous blocs, face intersectional vulnerabilities related to their class, religion, caste, sexuality, or disability status (Chakrapani et al., 2023; Huq et al., 2021; Riley et al., 2022), and are exposed to shared individual and environmental barriers to action, such as privacy norms, risk of backlash, or presence of violent crime, which we have described in prior work (Gram et al., 2023). We also stress that gender differences in affective response to VAW do not in any way imply ‘less rational behaviour’ among female participants. Emotion and rationality are not mutually exclusive categories and empathic emotions can in fact make behaviour more rational than pure self-interest when collective goods are at stake (Gram, Granados, et al., 2021b). Finally, we caution that it is beyond the scope of this study to place blame or seek accountability for the differences in men and women's engagement in addressing VAW. Instead, this paper seeks to argue that the structurally different positions of men and women result in socially constructed enablers and barriers to action that encumber NGO efforts to mobilise men relative to women.

Our study also has limitations. We did not collect data specifically on internal NGO dynamics shaping social interactions between staff and community volunteers, which may have shed further light on the behaviour of male and female volunteers. Male and female community members knew that the interviewer, female author XX, came from an NGO focused on preventing VAW and may have wanted to favourably impress her by expressing gender equal attitudes. NGO staff and female respondents cautioned us that men in the community would put on a pro-gender equality front whilst behaving differently out of sight. We addressed these concerns by triangulating results against discussions with NGO staff who routinely worked in the communities. We also supplemented qualitative data with quantitative data from SNEHA's monitoring system to strengthen our claim to gender differences in action to address VAW. Referral data were recorded by NGO counsellors rather than community members at the point of registration, which minimises recall and social desirability bias. Further, any desire among men to hide gender unequal views and behaviours would, if anything, would result in real gender differences being even sharper than those reported in this paper.

Nonetheless, our findings question the appropriateness of behaviour change techniques to overcome structural barriers to male engagement. For example, NGO efforts to circumvent barriers through alternative messaging have run into challenges. SNEHA tried to mitigate against men's perceived low stake in addressing VAW by focusing initial sessions on sexual health—what Casey et al. (2018) call conversation ‘hooks’—before discussing VAW. Although this attracted many young, single men to group sessions, they had difficulties connecting domestic violence to their own lived experience as they were not partnered. Further, SNEHA considered motivating men by promoting their empowerment and leadership but chose not to so as to avoid exacerbating male privilege. Inspired by the Yaari Dosti model (Verma et al., 2008), male volunteers were trained in allyship, but almost none of the men in our sample had ever worked with women on addressing VAW, nor could female respondents recall cases in which they had worked with male NGO members.

Community activities with men and women have similar costs for SNEHA, so if male participants contribute less to community prevention of VAW than women, this raises questions about the cost-effectiveness of funding male engagement rather than allocate funds towards work with women (Jewkes et al., 2015). Evaluations of male engagement interventions in India have found less evidence for impacts on behaviours such as bystander action compared to outcomes such as self-reported gender attitudes (Miller et al., 2014; Verma et al., 2008). This suggests that outward-facing activities such as promoting non-violence among one's neighbours may be more difficult to inspire than changes in male participants' own attitudes. Given sometimes undue pressure on VAW prevention programmes to engage men (Flood, 2015), it may be useful to recognize the value of programmes with differing levels of male engagement, including women-only programmes or programmes in which male community participants play a secondary role and are not expected to be as active as female participants.

In light of structural barriers to men's participation in community action to address VAW, it may be pertinent to revisit the role of NGOs. Neoliberal conceptions assign NGOs a role as nimble service providers addressing issues neglected by states impeded by inefficient and inflexible bureaucracies (Gupta & Sharma, 2006). Although this argument may have merit in contexts of weak state capacity, it should not be overvalued to the extent that NGOs become imagined as the *only* vehicle for addressing issues such as VAW (Roy, 2015). Dilemmas of how to allocate limited funding between activities engaging men, as opposed to women, when greater intensity of effort and investment may be required to inspire men, derive uniquely from a position of trying to solve the problem of VAW whilst relying almost exclusively on NGOs. Whole-of-society efforts to address VAW involving policies targeting institutions and systems at multiple levels, from media and politics to education and employment, could tackle the structural barriers that perpetuate gender inequality, including inequalities in community participation between men and women, in the first place. However, making such efforts a realistic prospect may require us to reject ‘NGOized’ perspectives on VAW prevention and imagine alternative futures.

## CRediT authorship contribution statement

**Sukanya Paradkar:** Writing – review & editing, Project administration, Investigation, Formal analysis, Data curation. **Chatush Singh:** Investigation. **Anand Suryavanshi:** Investigation. **Apurva Tiwari:** Investigation, Data curation. **Beniamino Cislaghi:** Writing – review & editing. **Nayreen Daruwalla:** Writing – review & editing, Supervision. **David Osrin:** Writing – review & editing, Supervision. **Lu Gram:** Writing – review & editing, Writing – original draft, Supervision, Methodology, Formal analysis, Data curation, Conceptualization.
